# Establishing consensus on key public health indicators for the monitoring and evaluating childhood obesity interventions: a Delphi panel study

**DOI:** 10.1186/s12889-020-09814-y

**Published:** 2020-11-17

**Authors:** Shane O’Donnell, Gerardine Doyle, Grace O’Malley, Sarah Browne, James O’Connor, Monica Mars, M-Tahar M. Kechadi

**Affiliations:** 1 School of Sociology, University College Dublin, D04 V1W8, Dublin, Ireland; 2 UCD College of Business and UCD Geary Institute for Public Policy, University College Dublin, Dublin, A94 XF34 Ireland; 3 School of Physiotherapy, Division of Population Health Sciences, Royal College of Surgeons Ireland, D02 YN77 Dublin, Ireland; 4 Children’s Health Ireland, Temple Street, D01 XD99 Dublin, Ireland; 5 School of Public Health, Physiotherapy & Sports Science, Woodview House, Belfield, University College Dublin, Dublin, 04V1W8 Ireland; 6 School of Computer Science, Insight Centre for Data Analytics, University College Dublin, D04 V1W8 Dublin, Ireland; 7 Division of Human Nutrition and Health, Wageningen University and Research, PO Box 17, NL-6700 AA Wageningen, The Netherlands

**Keywords:** Delphi, Obesity, Intervention, Childhood obesity, Evaluation, Public health, Health policy

## Abstract

**Background:**

Childhood obesity is influenced by myriad individual, societal and environmental factors that are not typically reflected in current interventions. Socio-ecological conditions evolve and require ongoing monitoring in terms of assessing their influence on child health. The aim of this study was to identify and prioritise indicators deemed relevant by public health authorities for monitoring and evaluating childhood obesity interventions.

**Method:**

A three-round Delphi Panel composed of experts from regions across Europe, with a remit in childhood obesity intervention, were asked to identify indicators that were a priority in their efforts to address childhood obesity in their respective jurisdictions. In Round 1, 16 panellists answered a series of open-ended questions to identify the most relevant indicators concerning the evaluation and subsequent monitoring of interventions addressing childhood obesity, focusing on three main domains: built environments, dietary environments, and health inequalities. In Rounds 2 and 3, panellists rated the importance of each of the identified indicators within these domains, and the responses were then analysed quantitatively.

**Results:**

Twenty-seven expert panellists were invited to participate in the study. Of these, 16/27 completed round 1 (5 9% response rate), 14/16 completed round 2 (87.5% response rate), and 8/14 completed the third and final round (57% response rate). Consensus (defined as > 70% agreement) was reached on a total of 45 of the 87 indicators (49%) across three primary domains (built and dietary environments and health inequalities), with 100% consensus reached for 5 of these indicators (6%).

**Conclusion:**

Forty-five potential indicators were identified, pertaining primarily to the dietary environment, built environment and health inequalities. These results have important implications more widely for evaluating interventions aimed at childhood obesity reduction and prevention.

## Background

Childhood obesity is considered one of the key public health challenges of the twenty-first century, with worldwide prevalence having increased from < 1% in 1975 to 6–8% in 2016 [1]. Since the publication of the influential *Foresight Tackling Obesities: Future Choices* report, the aim of which was to use the scientific evidence base from multidisciplinary research to identify the broad range of factors that influence obesity [2], policymakers have been increasingly encouraged to adopt a ‘system-level approach’ in their efforts to design and implement public health interventions to address childhood obesity. This acknowledges that the causes of obesity and overweight are multiple and complex, and the development of effective interventions is dependent on addressing myriad determinants rooted simultaneously in individuals’ biology as well as the socio-ecological conditions in which they grow up, learn, play, and work [3–5]. A significant gap in current literature is a socio-ecological inventory of factors important in the aetiology of childhood obesity, that simultaneously provides an indication of the relative weighting of factors in terms of priority for intervention. It is anticipated that such an inventory may be important for the monitoring and evaluation systems that collect multiple sources of data with a systems-level framework. Thus, the aim of this study was to identify indicators pertaining to childhood obesity that should be prioritised in the monitoring and evaluation of childhood obesity interventions. This study was conducted as part of a wider EC H2020 funded project entitled *Big Data Against Childhood Obesity* (BigO) which aims to develop a technology system that leverages the potential of big data to support public health authorities in formulating effective, context-specific policies and interventions addressing childhood obesity [4, 6, 7]. An indicator can be broadly defined as a measure that reveals relative positions in a given area (e.g. health). When evaluated at regular intervals, an indicator can point out the direction of change in different populations and across time [8, 9]. Choosing an appropriate set of indicators relevant to the monitoring and evaluation of health interventions requires a high degree of judgement and consensus-building among potential users and other interested parties [10]. Given the large number of variables that influence obesity at a system level (the Foresight report identifies over 100), and therefore the multiplicity of indicators that could be seen as a priority, a Delphi approach was adopted to reach consensus amongst an international panel of public health experts [11].

## Methods

### Study design

The Delphi process utilises a range of qualitative and quantitative approaches to reach decision-making among isolated anonymous respondents and is considered a valid methodology for obtaining a collective view from a group of experts where the only alternative is entirely subjective or based on anecdotal evidence [11, 12]. Delphi guides expert group opinion towards a final decision through triangulation of subjective group judgments [13] and is achieved through the application of **five** core features: (i) anonymity; (ii) iteration (iii) controlled feedback; (iv) statistical group response, and (v) expert input [9, 12]. In this study, we attempt to capture a broad set of indicators in the domains of the dietary environment, built environment and health inequalities. Following other studies that have adopted a Delphi approach for policy planning [14], the research involved five phases: indicator screening and categorisation; recruitment; evaluation; re-evaluation; and final consensus. A summary of the procedural steps taken in this Delphi panel study is as follows: firstly, a list of indicators was identified and categorised by qualitatively analysing initial pilot interviews conducted with experts in the public health sector in Ireland, Greece, and Sweden. Three broad domains of interest were identified, including the built environment, dietary environment and health inequalities. Then, in the first round of the Delphi study, a larger panel of experts was recruited and asked to identify the indicators that were most relevant for the design (and subsequent evaluation) of future policies aimed at reducing childhood obesity in these domains. These indicators were then analysed quantitatively over two rounds. In the second round, participants were asked to give importance ratings for each indicator. Responses were then quantitatively analysed to establish the level of consensus agreement for each indicator. Finally, in the third round, the panel of experts were made aware of the consensus levels of each indicator and asked whether they would like to change their response based on this finding. After importance ratings were given for each indicator, a final level of consensus was established. This process is described in more detail in the following section.

### Survey development

This study followed a traditional 3-round decision-making Delphi panel study [9, 15]. In order to identify the domains of interest that would feature in the Delphi panel, an initial pilot study was carried out with PHAs in Ireland, Sweden and Greece. These countries were selected because they were represented within the BigO research consortium. A list of indicators was drafted based on the indicators deemed of particular relevance to childhood obesity outlined in the Foresight report and Systems Map to help guide the discussion [2] (Table 1). Given that the PHAs time to participate was limited, some indicators had to be omitted, so we chose those we felt would be most relevant to PHAs. During the pilot stage, the PHAs were asked to review and comment on the relative importance of each indicator. We also sought their opinion regarding indicators that were missing. The first pilot interview was conducted face-to-face with a PHA based in Ireland. The same list was then discussed with PHAs in Sweden and Greece via video call. This process helped to further refine the list of indicators by eliminating those that were deemed less relevant to the PHAs. For example, the domain related to individual-level satiety control was deemed not as relevant to PHAs. Three authors (SO’D, GO’M, and SB) analysed the responses to identify themes and propose statements. The outcome of this phase was the identification of a number of areas of interest which could be categorised into three main domains:

- Built environment
- Dietary environment
- Health inequalities

**Table 1:** Indicators presented to PHAs as part of initial pilot interviews

Domain	Measurable Influencing Factors	Measurable Influencing Factors (cont.)
**Physical Activity**		
	Gender	Walkability
	Age	Urban planning
	BMI Grouping	Sedentary time
	Disability	Accessibility
	Prevelance of inactivity	Time and type
	Inequality	Affordability
**Social Environmental**		
	Water quality	Green space
	Air quality	Food waste
	Proximity to motorway	School policy
	Density of food retailers	Organic pollutant
	Location of food retailers	Weather pattern
	Food desserts	Advertising/marketing density
	Psychosocial distress	Stigma
**Financial**		
	House price	Disposible income
	Homeownership	Household food spend
	Area deprivation	
**Individual level**		
	Medical history	Genetics
	Health service utilisation	Family history

Based on this outcome, a set of questions was developed to be used in the first round of the Delphi survey. These questions were initially piloted amongst a small number of PHAs in Ireland and Sweden (*N* = 3) to ensure that they were congruent, easy to understand and culturally appropriate (Table 2).

**Table 2:** Delphi Panellists

Country	Key panellist type	***N*** =
Sweden		4
	Academic	
	Policy maker	
Greece		2
	Academic	
	Policy maker	
Ireland		7
	Academic	
	Policy maker	
	Public health advocate	
Netherlands		1
	Policy maker	
Spain		1
	Academic	
United Kingdom		1
	Academic	

### Recruitment & Data Collection

A purposeful stratified sampling technique was used to identify potential panellists [16]. Following Novakowski and Wellar (2008), strict criteria for the selection of expert panellists was developed to include only those with:

- Direct influence over policy at both local and national levels
- Indirect impact by shaping policy through scholarly research and public advocacy

Following Keeney et al. [11], we aimed to recruit a total of 15–20 panellists. A panel of experts was identified and 27 invitations to participate were sent to PHAs in Ireland, Sweden, Greece, the Netherlands, Spain and the UK. The countries selected for participation (with the exception of the UK) reflected where the organisations involved in the H2020 BigO project were based and also where the BigO system would initially be rolled out. Thus, we were able to leverage the local knowledge and extensive networks of the BigO research teams in each of these jurisdictions to purposefully identify individuals with the requisite expertise. Of those who were invited, 16 initially agreed to take part. Surveys were then distributed to panellists via the online survey Qualtrics™ in English only. In the first round, panellists were given the freedom to respond to each of four questions in narrative form and encouraged to elaborate on their responses in an in-depth manner.

### Data analysis

#### Round 1

Once the 16 responses were returned content analysis was performed. Each response was analysed line-by-line to identify distinct statements made by panellists that related to measures or indicators [11]. Subsequently, statements similar in nature were grouped together under one ‘prototypical’ statement to reduce the size of the subsequent questionnaires and ease the burden on panellists in completing later rounds [11]. One issue that emerged at this stage of the analysis was that the term ‘measure’ in each question was interpreted by some to mean ‘actions to be taken’ rather than specific indicators or measures of progress. As such, the wording of each statement was changed to avoid further confusion or ambiguity in subsequent rounds. The result was the generation of a list of 87 statements, which remained constant in all subsequent rounds.

#### Round 2

Each statement was uploaded onto Qualtrics and panellists were invited to rate the relative importance of each statement using a 5-point Likert scale. Panellists were given 3 weeks to submit their responses. Regular reminders (*N* = 3) were sent to those who had not yet completed the survey. A predetermined level of consensus, known as the percentage agreement, was set at ≥70%. Only indicators rated as either ‘very’ or ‘extremely important’, by at least 70% or more of the panellists, were deemed to have reached consensus [6, 17–20]. The results of Round 2 were analysed quantitatively using the Software Package for Social Science (SPSS) [21], to calculate the central tendency (Mean, Median, Mode) and level of dispersion (Standard Deviation), in order to present information concerning the collective judgements of respondents in Round 3.

#### Round 3

We retained all indicators from Round 2 so that each indicator had an equal opportunity to gain the highest rating of importance possible [11]. Panellists were asked to rate the same statements as presented in Round 2. However, each statement was accompanied by two additional pieces of information: the rating the individual panellist assigned to each statement in the previous round, as well as the average response of the group (mode). Panellists were then invited to consider if they would like to change their response in light of the aggregate opinion of other panellists or stand by their original response. Finally, Round 3 was analysed quantitatively using descriptive statistics as in Round 2.

## Results

Of the 27 experts invited to participate in this Delphi study, 16 completed round 1 (59% response rate), 14 completed round 2 (87.5% response rate) and 8 completed round 3 (57% response rate). Table 4 below shows a summary of the statements and the level of consensus achieved in each domain. In Round 1, there was no consensus level, as this round was designed to establish the indicators in each domain. In Round 2, 43 of the 87 indicators (49%) passed the consensus agreement threshold (Table 3). This rose to 45 indicators (52%) in Round 3, with variation in the individual indicators that reached consensus between rounds 2 and 3.

**Table 3:** Summary of the number of indicators that reached consensus agreement in each round by domain

Statement domains	Number of statements in each domain			Proportion of statements where consensus was achieved (***n***)		
	Round 1	Round 2	Round 3	Round 1	Round 2	Round 3
Built environment		34	34		47% (16)	47% (16)
Dietary environment		30	30		40% (12)	50% (15)
Health inequalities		19	19		74% (14)	68% (13)
Uncategories		4	4		25% (1)	25% (1)

**Fig. 1:**
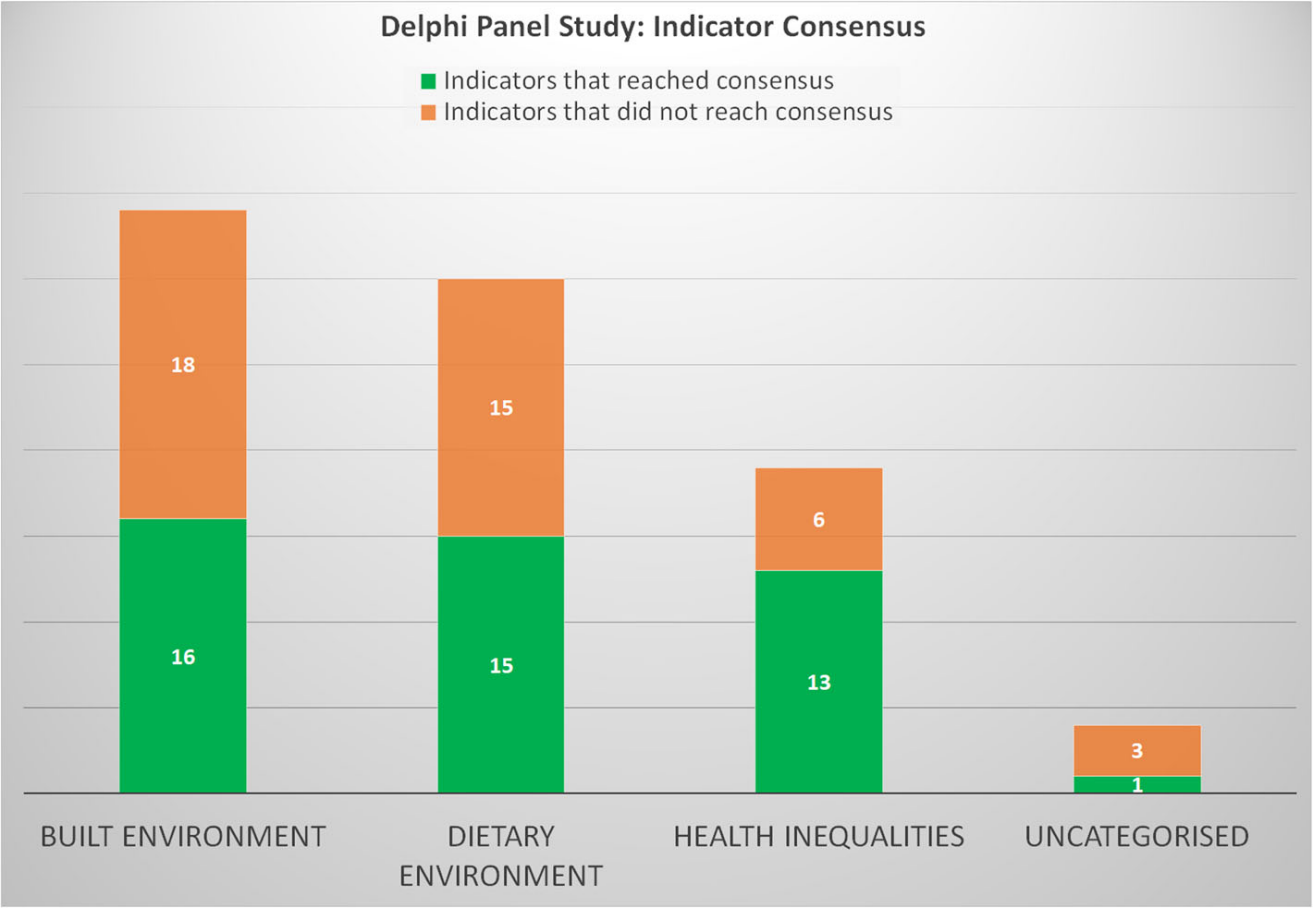
Delphi Panel Study: indicator consensus

Additionally, in Round 3, 100% consensus was reached for some indicators in the **Built Environment** domain (*n* = 2), **Dietary Environment** domain (*n* = 2), and **Uncategorised** domain (*n* = 1), with no indicator reaching full consensus (consensus range 87.5–0%) in the **Health Inequalities** domain (Table 4). Stability of consensus (< 10% variation) was achieved between rounds 2 and 3 for all four domains [6]. Of the remaining indicators, 12 were just below the percentage agreement level (70%), reaching a consensus level of 62.5%. From the five indicators that reached complete consensus (100%), two were indicators that relate to the school environment, one economic (food prices), one governmental (cycle lanes) and one personal health (BMI). For the remainder of indicators that passed consensus threshold, more than half were related to government resources in a given environment (access to facilities or the lack thereof and exposure to unhealthy food sources and their advertisements), economic inequalities, and school structures (access to facilities and healthy food). Indicators that were least likely to reach consensus related to access and affordability of alcoholic beverages (availability of off-licences/liquor stores and minimum alcohol unit pricing). A graph showing the numbers of indicators that reached consensus is also shown [Fig. 1].

**Table 4:** List of indicators and results (Arranged Thematically) - Indicator consensus after 3 rounds of the Delphi Study degree of consensus

Domains	Question for each domain	SD
Built Environment	In the design (and subsequent evaluation) of future policies aimed at improving the built environment to reduce childhood obesity what, in your opinion, are likely to be the most useful measures in your jurisdiction?	
Dietary Environment	In the design (and subsequent evaluation) of future policies aimed at improving the dietary environment to reduce childhood obesity what, in your opinion, are likely to be the most useful measures in your jurisdiction?	
Health Inequalities	In the design (and subsequent evaluation) of future policies aimed at improving inequalities in childhood obesity-related outcomes what, in your opinion, are likely to be the most useful measures in your jurisdiction?	
Uncategorised	Are there are any other measures related to childhood obesity prevention and monitoring that you feel are important to capture at community/population levels?	
**Measurements**	**Degree of Consensus (%) Round 3**	
**Built Environment**		
Q1_36 School infrastructure that includes spaces for organized or individual exercise/activity	100%	0.5
Q1_57 Availability of safe cycling paths	100%	0.34
**Dietary Environment**		
Q2_28 The pricing environment of foods	100%	0.5
Q2_42 Availability of tap water in schools	100%	0.43
**Uncategorised**		
Q4_24 BMI changing over time in terms of mean, median and shape of distribution	100%	0.43
**Built Environment**		
Q1_31 Availability of outdoor facilities	87.50%	1
Q1_35 Recreational space within walking space of distance of home	87.50%	0.71
Q1_39 Availability of open spaces in neighbourhood.	87.50%	0.35
Q1_41 Density of public parks	87.50%	0.7
Q1_42 Proximity of green space to home	87.50%	0.71
Q1_47 Design of walkways and physical environment	87.50%	0.71
**Dietary Environment**		
Q2_29 Range and diversity of food retailers	87.50%	0.66
Q2_30 Number of fast food advertisements within the community	87.50%	0.66
Q2_34 Digital exposure to food advertising	87.50%	1.2
Q2_35 Availability of fresh fruit and vegetables	87.50%	0.7
Q2_36 Retail environment within supermarkets	87.50%	1.2
Q2_38% of taxes on sugar	87.50%	0.66
Q2_39% of taxes imposed on foods high in fat and salt	87.50%	0.66
Q2_41 Availability of healthy meals in school and preschool	87.50%	1
Q2_54 Infant feeding indicators	87.50%	0.73
**Inequalities**		
Q3_9 Employment status or socio-economic status of family	87.50%	1
Q3_10 Local deprivation indices	87.50%	0.99
Q3_11 Area based food poverty statistics	87.50%	0.97
Q3_12 Number of households experiencing food poverty	87.50%	0.99
Q3_13 Unemployment levels	87.50%	0.99
Q3_14 Child and family – Living on public assistance	87.50%	0.97
Q3_16 Ethnicity	87.50%	0.87
Q3_18 Family structure	87.50%	0.5
Q3_22 Relative income poverty in line with government measures on inequality	87.50%	0.7
Q3_23 Consistent poverty in line with government measures on inequality	87.50%	0.7
Q3_24 Deprivation in line with government measures on inequality	87.50%	0.71
Q3_25 Additional metrics of social inequality used both individually and as components of census-derived, weighted, area-level deprivation indices	87.50%	0.7
**Built Environment**		
Q1_37 Affordability of organized sports: club fees and costs	75%	0.71
Q1_40 Number of public parks	75%	1.1
Q1_44 Availability of public transport to access green spaces	75%	0.71
Q1_49 Sports and physical activity participation levels	75%	1.1
Q1_51 Opening hours of green spaces	75%	0.71
Q1_52 Quality of lighting within green spaces	75%	0.8
Q1_53 Level of reported anti-social behaviour in green spaces / open spaces	75%	1
Q1_56 Accessibility of public transport via foot	75%	1.05
**Dietary environment**		
Q2_24 Density and type of food retailer in proximity to school	75%	0.83
Q2_31 Advertisements in proximity of schools	75%	2
Q2_40 Availability of High Fat Salt Sugar foods/drinks	75%	0.73
Q2_46 Availability of energy-dense foods in vending machines and cafeterias in the school environment.	75%	1.1
**Inequalities**		
Q3_8 Education level statistics	75%	1.3
**Built Environment**		
Q1_27 Availability of indoor facilities	62.50%	0.86
Q1_30 Price of indoor facilities	62.50%	1.1
Q1_34 Price of outdoor facilities	62.50%	1.1
Q1_50 Accessibility of public parks via public transport	62.50%	0.8
Q1_55 Access facilities for fitness training at no cost to the individual	62.50%	1.3
Q1_60 Number of cars located on the road outside home	62.50%	1.3
**Dietary environment**		
Q2_37 Location where children do their shopping	62.50%	1.2
Q2_45 Data on the range and quality of food served in the workplace settings	62.50%	1.1
**Inequalities**		
Q3_15 Health literacy	62.50%	1.3
Q3_20 Availability and access to school meals schemes	62.50%	1.05
**Uncategorised**		
Q4_26 Monitoring of diets of families/children considered at risk by social workers/social services	62.50%	1.3
Q4_27 Whole production chain needs to be attended to	62.50%	1.12
**Built Environment**		
Q1_38 Numbers of people who use recreational spaces	50%	1
Q1_43 Proximity of blue space to home	50%	0.5
Q1_48 Child and parental attitudes and knowledge of their built environment	50%	0.87
Q1_62 Number of physical activity referrals / prescriptions in general practice	50%	1.2
**Dietary environment**		
Q2_26. Density and type of food retailer along school commute	50%	
Q2_27 Tracking data on portion sizes in fast-food retailers, other restaurants and single-serving snacks	50%	0.83
Q2_33 Food advertising at specific times	50%	1.1
Q2_50 Exposure to alcohol advertising at sporting events	50%	1
Q2_52 Availability of café/bars	50%	1.05
Q2_44 Access to community gardens	42.90%	0.99
**Inequalities**		
Q3_17 Gender	42.90%	0.83
Q3_19 Availability and access to universal primary health services	42.90%	1.25
**Built Environment**		
Q1_29 Density of indoor facilities	37.50%	1.2
Q1_32 Number of outdoor facilities	37.50%	1.3
Q1_33 Density of outdoor facilities	37.50%	1.1
Q1_58 Availability of walk to school groups	37.50%	0.86
**Dietary environment**		
Q2_25 Density and type of food retailer in proximity to home	37.50%	0.71
Q2_32% of processed food items with clear and accurate front of pack labelling	37.50%	1
Q2_43 Access to allotments- % of school with allotments	37.50%	1.1
Q2_48 Minimum alcohol unit pricing	37.50%	0.97
**Inequalities**		
Q3_26 Availability of cooking and growing skills programmes	37.50%	1.05
**Uncategorised**		
Q4_25 Provide special support to individual cases	37.50%	0.66
**Built Environment**		
Q1_46 GIS based measures of cyclability	25%	0.6
**Dietary environment**		
Q2_51 Alcohol use as a contributor to adolescent obesity	25%	0.78
Q2_53 Access to farmers markets	25%	1.17
**Built Environment**		
Q1_45 GIS based measures of walkability	14.30%	0.64
Q1_28 Number of indoor facilities	12.50%	0.93
Q1_59 Availability of Park and Ride schemes	12.50%	1.1
**Dietary environment**		
Q2_47 Availability of off-licences/liquor stores	12.50%	1.1
**Inequalities**		
Q3_21 Level of referrals to GP	0%	0.7

## Discussion

This study engaged with PHAs and advisors to identify and prioritise indicators deemed important for the monitoring and evaluation of childhood obesity interventions. In our study, Consensus (defined as > 70% agreement) was reached on a total of 45 of the 87 indicators (49%) across three primary domains (built and dietary environments and health inequalities), with 100% consensus reached for 5 of these indicators (6%). The consensus reached in a large number of factors underscores the level of complexity involved in obesity intervention and the challenges implementing change in these domains.

With certain exceptions [6, 22–24], few studies have explored indicators relevant to the development and monitoring of childhood obesity-related policies. One study employed the Delphi panel technique and focused on the prioritisation of intervention conditions in childhood obesity [25]. Others have focused on research priorities among clinical and academic experts [22, 26]. For example, the Determinants of Nutrition and Eating framework (DONE) study, which employed a three-round Delphi panel study to examine the priorities of policymakers with respect to healthy eating, identified a similar set of indicators with respect to the dietary environment to those elucidated in this study [22]. Our study further builds on this work by addressing other domains deemed important by PHAs - the built environment and wider inequalities related to childhood obesity and includes a number of PHA from a variety of different countries with contrasting health policies. Interestingly, given the growing awareness of the role that the social determinants of health play in the aetiology of childhood obesity, it is perhaps somewhat unexpected that there was not 100% consensus for any indicator within the health inequalities domain [4]. However, it is also worth noting that although 100% consensus was not reached, it did have the highest rate of consensus (74% = 14 out of 19 indicators with a range of 87.5 to 0% consensus) compared to other domains and their indicators. Furthermore, those indicators pertaining to health inequalities that reached consensus were more likely to relate to wider patterns of social and economic inequality (e.g. unemployment, local deprivation indices, etc.,.). In contrast, many of the indicators in this domain that failed to reach consensus were centred on interventions that rely on a greater degree of personal agency and individual-level action (e.g. access to cooking and growing schools programmes, access to community gardens, availability and access to universal primary care services etc.). This is perhaps a reflection of a growing awareness amongst policy makers that while individual-level interventions may be helpful in improving overall population health, they may be less effective in reducing relative health inequalities [5].

This study has a number of important implications. First, the results highlight the variety and range of data that would be relevant to PHAs and the identification of indicators across multiple domains and underscores the system-based focus of PHAs in Europe. As outlined in the Foresight report, there are over 100 factors that contribute to childhood obesity and these are often interdependent (e.g. lower pricing of energy-dense food, excessive marketing of energy-dense food and excessive consumption of energy-dense food by children). Despite high-quality, international and longitudinal research programmes relevant to childhood obesity, integrating and monitoring multi-level system factors still presents a challenge. The Childhood Obesity Surveillance Initiative (COSI) from the World Health Organisation (WHO), for example, collects individual-level data on anthropometry, dietary and physical activity patterns, screen time, and sleep, among others. Recent findings from 6 to 9-year-olds in the WHO European Region demonstrates substantial country-level differences in healthy and unhealthy dietary habits, with patterns that cannot be fully explained [27]. With respect to physical activity, the Health Behaviour in School-Aged Children (HBSC) Study reports substantial variation in physical activity participation among school-aged children from 29 countries. National differences in the physical, socio-cultural, economic and policy environment account for individual-level differences [28]. Our study will complement existing knowledge and help to guide researchers who wish to further integrate socio-ecological factors into monitoring systems. Furthermore, our findings are particularly timely as they can help to guide the development of emerging big data solutions for the monitoring and surveillance of ongoing efforts to reduce and prevent childhood obesity through a systems approach [4, 6, 7].

A second implication drawn from our findings is the importance of co-developing monitoring systems in collaboration with policy makers and other relevant stakeholders who have good knowledge of their own local context and the data repositories required or in some cases already available for use. For example, to help evaluate a policy intervention on childhood obesity prevention, such as regulating the distance a fast-food retailer could be built in proximity to a school, multiple related data sources are needed across the domains identified in this study. To monitor change over time and evaluate the effects of this intervention, data is needed at the level of the child, at the level of the school and at the community level. To successfully source, store, retrieve, analyse, and present the socioeconomic-, health-, dietary, economic- and geospatial data needed in the above example, a number of considerations are required to meet legal, data protection, privacy and ethical requirements in addition to the necessary standards, protocols and technological aspects. Monitoring and surveillance systems need to address these concerns so that public health officials can evaluate and monitor the effectiveness of such interventions on childhood obesity and so that decision-making can be facilitated for scaling up successful interventions. However, it is also important to acknowledge that obesity is the outcome of a complex adaptive system and the success of any intervention is dependent on the wider social context in which it is deployed and embedded. As one panellist pointed out, exclusive reliance on quantitative analysis of macro-level indicators (regardless of the sophistication of the predictive models used) may be too reductionist to provide the holistic picture needed to understand the nuances of this complex adaptive system. Future research must therefore examine how best to incorporate both quantitatively driven, macro-level indicators and qualitative data, which is more appropriate for capturing the wider context and lived experiences of children or communities for whom obesity interventions may be implemented.

### Strengths and weaknesses

Through the use of the Delphi method [12–15, 29], this study captured the collective feedback [*N* = 16] from some of the leading obesity experts in Europe across a wide range of European regions with varying health systems. For example, based on the European Core Health Indicator of Expenditure on health care as a percentage of GDP in 2018, Sweden (10.90) and the Netherlands (9.97) are above average while Ireland (6.93) and Greece (7.72) are below average [30].

Consensus was found across a wide range of statements which in turn enabled the research team to delineate a list of indicators which can be used to improve and inform the further development of systems to monitor and evaluate ongoing public health efforts to reduce and prevent childhood obesity. Many of these indicators, particularly pertaining to the built environment, have yet to be prioritised in the extant literature. In addition, the study also highlights where there is perhaps less certainty among policy makers (e.g. indicators pertaining to health inequalities) and therefore areas for further inquiry.

While a key strength of the study is it’s leveraging of expert knowledge of some of the leading authorities with a remit in childhood obesity prevention in Europe with a wide variation in health policy, recruitment of panellists was nonetheless limited to 6 countries and, as such, their perceptions, understandings and insights may not be generalisable to all European countries or to jurisdictions outside Europe. Furthermore, the recruitment relied on the networks of the BigO research team, and therefore, some element of bias in the selection of participants at both the piloting and main data collection phases cannot be ruled out. Another issue was the diminished response rate between rounds, with half the participants lost between Rounds 1 & 3. Given that Delphi panel studies rely on two or more iterative rounds, the content of which can often be repetitive, an attrition rate of up to 50% is not uncommonly reported in the literature [31]. One possible explanation for the attrition in this context of this study may have been the onerous nature of assessing large numbers of statements in each round. It has been noted that in instances where Delphi panel studies include a high number of items, panellists are less likely to participate all the way through to study completion [32]. Given many of these participants would have been leading authorities in their respective jurisdictions, their time to complete the survey would have been limited. Whilst the research team were conscious of this risk from the outset of the study, the importance of minimising the burden of participants also had to be balanced against the need to ensure that the final list of indicators was as comprehensive as possible to reflect the complexity of childhood obesity-related policy.

Nevertheless, the final sample size [*N* = 8] sits between what the NIHR Health Technology Assessment group [33] identifies as the lower threshold for participation in consensus groups at which point validity begins to decline rapidly [*N* = 6], and the upper threshold at which point any improvements in validity may become subject to diminishing returns [*N* = 12]. Indeed, there is little existing theoretical or empirical evidence that increasing larger sample sizes in Delphi studies necessarily leads to more reliable or valid results [6, 34–38].

In addition to examining the robustness of the indicators identified in this study among PHAs based in other jurisdictions, future studies should also incorporate the voices of other important stakeholders, such as supranational organisations (e.g. WHO); industry (e.g. insurance companies, device manufactures, marketing agencies); the healthcare sector; investors; non-health related government agencies; teachers, children and their parents. Furthermore, the development of co-designed tools and platforms, acceptability and usability studies (particularly focused on privacy and data sharing considerations) should be carried out to further inform how such systems might be used in practice by PHAs, researchers or citizens.

## Conclusion

This study contributes to current childhood obesity literature by providing expert consensus on a wide range of key socio-ecological influences and measures that are amenable to policy change, particularly in the areas of the built environment, dietary environment and health inequalities. Factors that should especially be prioritised include the school infrastructure that includes space for organised or individual exercise activity, availability of safe cycling paths, the pricing environment of foods, availability of tap water in schools and BMI changes over time. The volume and complexity of pertinent measures that should be collected require the implementation of smart technology solutions. The findings, therefore, have implications both in informing childhood obesity interventions and in developing systems that can monitor and evaluate those efforts.

## Data Availability

The datasets generated and/or analysed during the current study are not publicly available to protect the anonymity of participants but are available from the corresponding author on reasonable request.

## Abbreviations

BMI: Body Mass Index
COSI: Childhood Obesity Surveillance Initiative
DONE: Determinants of Nutrition and Eating framework
EU: European Union
GO’M: Grace O’Malley
HSBC: Health Behaviour in School-Aged Children
PHA: Public Health Authority
SB: Sarah Browne
SO’D: Shane O’Donnell
SPSS: Software Package for Social Science
UK: United Kingdom
WHO: World Health Organisation
